# Large Cystic Lymphangiomas of the Neck: A Surgical Challenge

**DOI:** 10.7759/cureus.85542

**Published:** 2025-06-07

**Authors:** Pavithra Subramaniyan, S.M. Azeem Mohiyuddin, Divya N Jyothi, Sagayaraj A, Kouser Mohammadi, Krishnappa J, Sujatha Munireddy Papireddy, Adarsh A D

**Affiliations:** 1 Otolaryngology-Head and Neck Surgery, Sri Devaraj Urs Academy of Higher Education and Research, Kolar, IND; 2 Otolaryngology-Head and Neck Surgery, Sri Devaraj Urs Medical College, Kolar, IND; 3 Otolaryngology-Head and Neck Surgery, Mangalore Institute of Oncology, Mangalore, IND; 4 Pediatric Medicine, Sri Devaraj Urs Academy of Higher Education and Research, Kolar, IND; 5 Anesthesia, Sri Devaraj Urs Medical College, Kolar, IND; 6 Radiodiagnosis, Sri Devaraj Urs Academy of Higher Education and Research, Kolar, IND

**Keywords:** cystic hygroma, de serres classification, lymphangiomas, postoperative complications, recurrence, surgical excision

## Abstract

Background: Cystic hygroma, a congenital malformation of the lymphatic system, predominantly affects pediatric populations but can also present in adults, posing unique diagnostic and therapeutic challenges. This study aims to document the clinical and radiological extent and involvement of important neurovascular structures and also the outcome of patients treated for large cystic hygroma in the head and neck region.

Methods: A retrospective analysis of medical records was conducted for all 33 patients who underwent surgical excision of large cystic hygromas in our hospital. The data collected included patient age, sex, lesion location, anatomical stage (using the de Serres classification), morphological type (cavernous or macrocytic), surgical outcomes, postoperative complications, and recurrences.

Results: The study population comprised 22 (66.7%) pediatric patients (<18 years), with a 21 (63.6%) male predominance. According to the de Serres classification, 24 (72.7%) patients presented with stage III disease, involving both the suprahyoid and infrahyoid regions. Morphologically, lesions of 19 (57.5%) patients were cavernous, while 14 (42.4%) were macrocystic. Complete surgical excision was achieved in all patients. Postoperative complications occurred in eight (24.24%) patients, including transient injury to the marginal mandibular branch of the facial nerve in three (9.09%) patients and transient paralysis of the facial nerve in one (3.03%) patient. Recurrence was seen in four (12.12%) patients.

Conclusion: Cystic hygromas are congenital lymphatic malformations frequently involving the head and neck. Surgical excision remains the mainstay of treatment for large lesions, though it poses technical challenges due to proximity to vital neurovascular structures. With meticulous dissection, complications can be minimized. Sclerotherapy is effective for smaller lesions but is less suitable for extensive disease. Recurrence is uncommon following complete surgical excision.

## Introduction

Cystic hygroma, also referred to as cystic lymphangioma, is a benign congenital anomaly arising from the abnormal development of the lymphatic system [1]. This condition occurs when embryonic lymphatic vessels fail to establish proper connections with the venous system, resulting in the sequestration of lymphatic sacs that eventually develop into fluid-filled, multilocular cystic masses [2]. Most cystic hygromas occur in the cervicofacial region, accounting for 75-80% of lymphatic malformations due to embryological lymphatic tissue distribution [1].

The majority of cases are diagnosed during infancy or early childhood, with over 90% presenting before the age of two [3]. However, delayed presentations can occur in older children or even adults, particularly when the lesions are deep-seated, asymptomatic, or mistaken for other soft tissue masses [4,5]. Lymphangiomas are classified based on cyst size into macrocystic (≥2 cm), microcystic (<2 cm), or mixed types [5]. Macrocystic lesions are more amenable to surgical excision, whereas microcystic and mixed lesions often infiltrate surrounding tissues and are more challenging to remove completely [6]. Although histologically benign, large cystic hygromas can cause significant clinical morbidity. The mass effect on surrounding structures may lead to symptoms such as airway compromise, dysphagia, or venous congestion [7].

While surgical excision remains the primary modality for accessible lesions, challenges arise due to proximity to vital neurovascular structures, necessitating adjunctive options like sclerotherapy in selected cases [8-10].

Despite the availability of various treatment options, literature focusing on the surgical management of large cystic hygromas remains limited, especially in low-resource settings. This study aims to analyze the clinical spectrum, anatomical considerations, postoperative outcomes, and recurrence patterns associated with large cystic hygromas of the neck. A better understanding of these factors is crucial for guiding surgical decision-making, optimizing patient outcomes, and having protocols in both high- and low-resource healthcare environments.

## Materials and methods

Study design

This retrospective observational study was conducted in the Department of Otolaryngology at Sri Devaraj Urs Medical College, Kolar, India, a tertiary care teaching hospital located in a rural area from January 2000 to October 2024. 

Study population and sample size

This study included 33 patients diagnosed with cystic hygroma involving the cervical region who underwent complete surgical excision and had a minimum follow-up of three years with complete medical records. Patients were excluded from the study if they had major scars or contracture on the neck, had previously undergone unsuccessful sclerotherapy or surgical management at another institution, or had incomplete medical records.

Study measures

The study aimed (1) to document the clinical features, radiological findings, and intraoperative findings in patients operated for large cystic hygromas of the neck and (2) to analyze the outcomes of the above patients and document complications or recurrences, if any.

Ethics statement

Ethical clearance was received from the Central Ethics Committee of Sri Devaraj Urs Academy of Higher Education and Research (approval number: SDUAHER/KLR/R&D/CEC/S/PG/90/2024-25) to conduct the research.

Methodology

Thirty-three patients who met the inclusion criteria were included in the study. A non-probability, consecutive sampling technique was used to enrol all eligible patients during the study period, thereby minimizing selection bias. Patients who had complete medical case records and confirmed postoperative histopathological diagnosis and a minimum of three-year follow-up were selected. Clinical features at the time of presentation, the extent and findings on imaging, staging, and morphological classifications were documented. The surgery done and the extent of the lesion, as seen in surgical documents, were recorded. Outcomes were documented with regard to disease-free survival, complete recovery, recurrence, or complications, if any.

Demographic data were recorded, and the patients were categorized into groups below and above 18 years of age. Findings on imaging contrast-enhanced computed tomography (CECT)/magnetic resonance imaging (MRI) were documented with regard to the extent of lesion and proximity or encasement of important neurovascular structures and then classified into macrocystic, microcystic, or cavernous types. The de Serres classification system was used to stage cystic hygromas based on anatomical involvement.

All patients underwent complete surgical excision under general anesthesia, with approaches tailored to the lesion location. Intraoperative observations documented the lesion characteristics and dissection challenges. Regular postoperative follow-up was used to assess complications and recurrence. The data included wound healing, surgical site infection, and neurological and vascular injury. Recurrence was defined as the reappearance of the lesion, which was confirmed clinically or radiologically. Data were presented as frequency and percentage.

## Results

Among the 33 patients included in the study, 22 (66.7%) were below 18 years of age, and 11 (33.3%) were above 18 years of age. Our study included 21 (63.6%) male patients and 12 (36.4%) female patients.

According to the de Serres staging, most patients were in stage III (suprahyoid and infrahyoid) (24, 72.7%), followed by stage II (unilateral suprahyoid) (9, 27.27%). No patients were found in stages I, IV, or V. Based on morphological type, cavernous lesions were observed in 19 (57.5%) patients, whereas macrocystic lesions were observed in 14 (42.4%) patients. No patient had microcystic lesions (Table 1).

**Table 1:** Distribution of patients based on the de Serres anatomical staging and morphological classification of cystic hygroma (n=33)

Patient distribution by anatomical stage and morphology		n (%)
de Serres staging	Stage I: unilateral infrahyoid	0
	Stage II: unilateral suprahyoid	9 (27.27%)
	Stage III: suprahyoid and infrahyoid	24 (72.7%)
	Stage IV: bilateral suprahyoid	0
	Stage V: bilateral suprahyoid and infrahyoid	0
Morphological type	Macrocystic lesions	14 (42.4%)
	Cavernous lesions	19 (57.5%)
	Microcystic lesions	0

**Figure 1:**
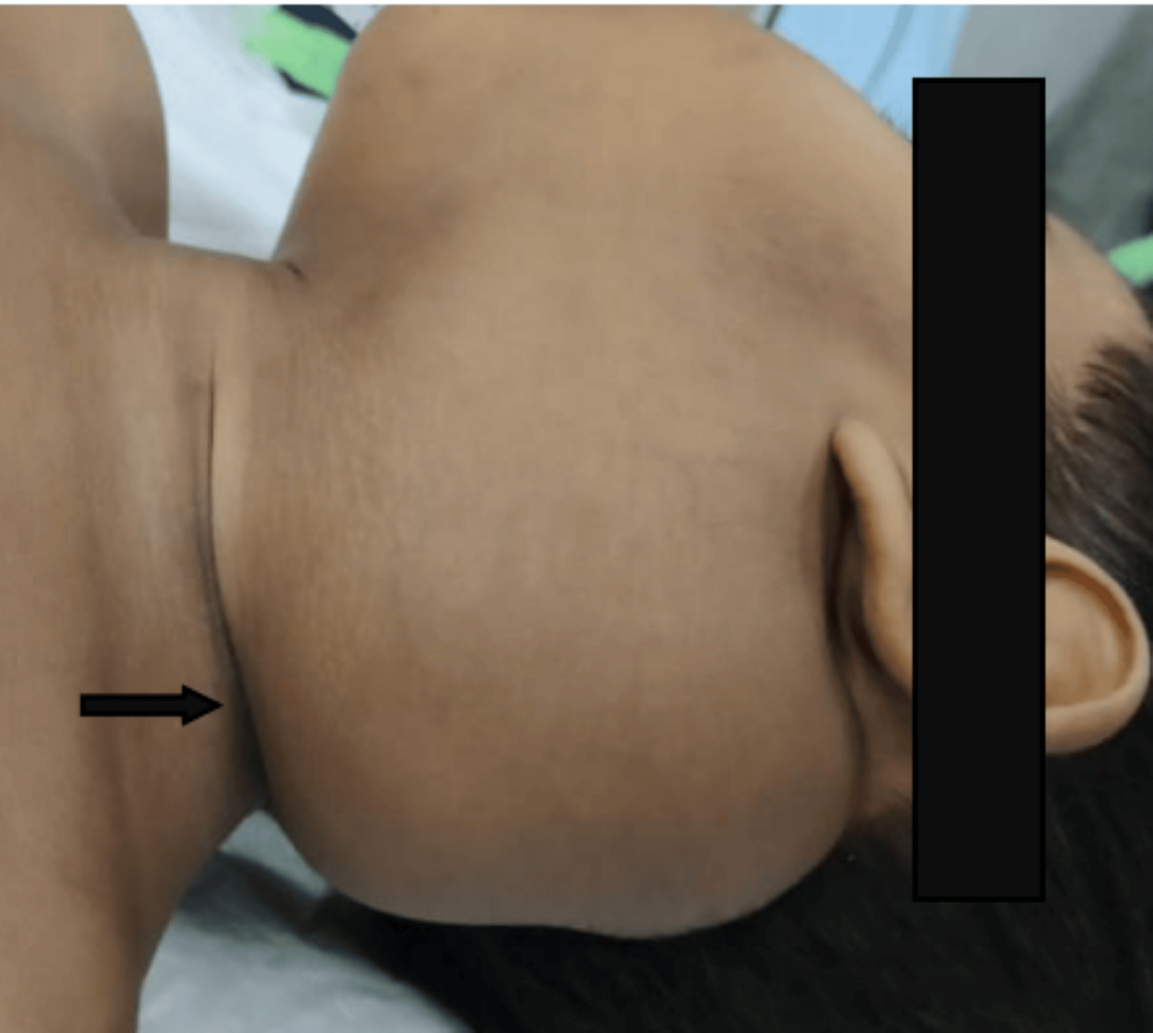
Preoperative clinical image of cystic hygroma in a pediatric patient This image shows a child presenting with a large, soft, non-tender, cystic swelling involving the left lateral aspect of neck, consistent with cystic hygroma.

A preoperative clinical image of cystic hygroma of the neck in a pediatric patient is illustrated in Figure 1. 

**Figure 2:**
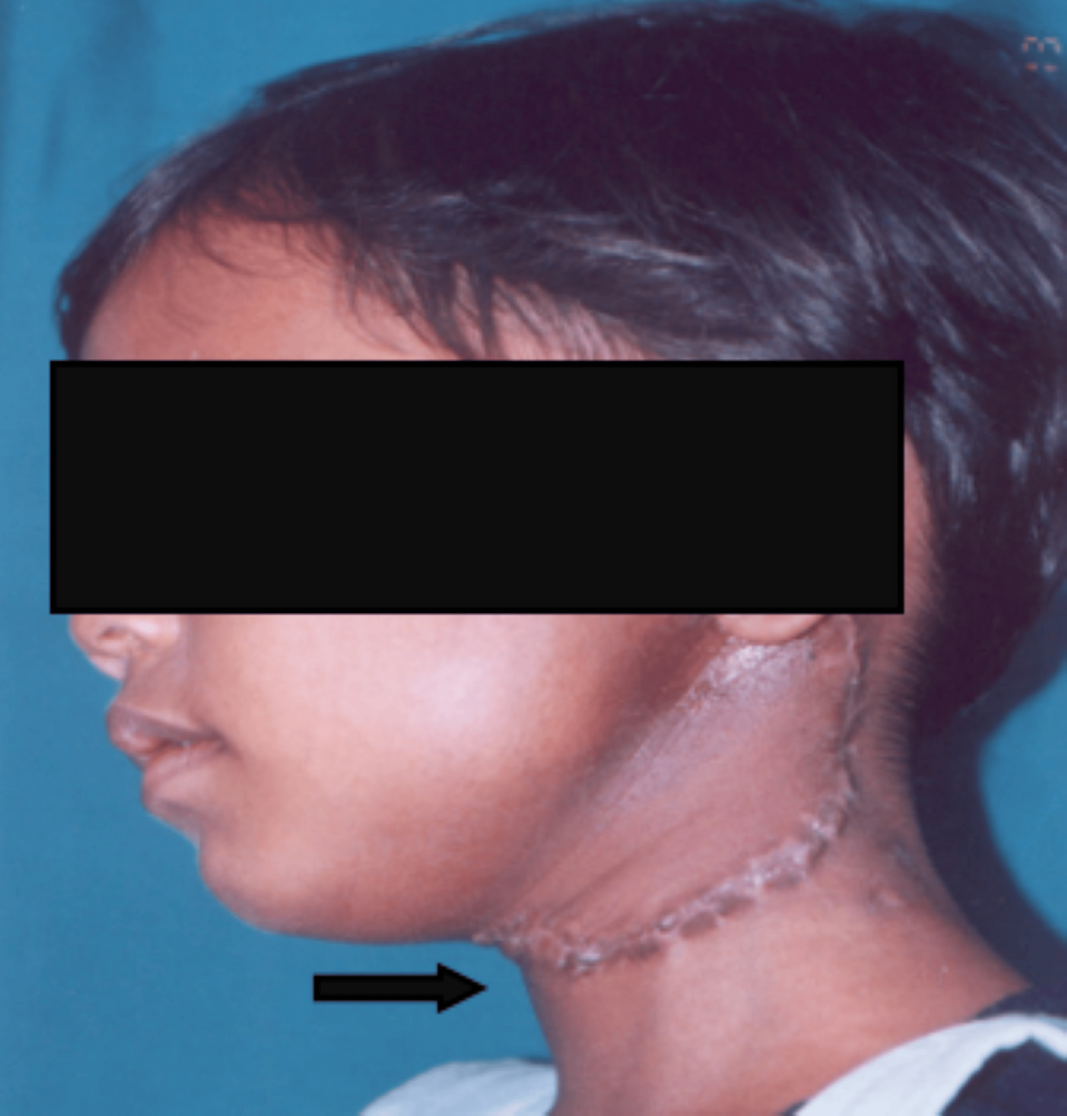
Surgical scar in the neck This image shows a lateral profile of a pediatric patient demonstrating a linear postoperative scar over the left side of the neck following the complete surgical excision of cystic hygroma.

A patient operated for a large lymphangioma extending to the parotid region with a postoperative scar in the neck is illustrated in Figure 2. 

Injury to the marginal mandibular branch of the facial nerve was the most common complication, reported in three (9.09%) patients, and recovered spontaneously within four months. Wound dehiscence in the infra-auricular region requiring secondary suturing occurred in two (6.06%) patients. Injury to the internal jugular vein, transient paralysis of the entire facial nerve (House-Brackmann grade IV) which recovered completely in six months, and neuropraxia of the spinal accessory nerve were each observed in one (3.03%) patient and recovered completely in four months (Table 2).

**Table 2:** Incidence and types of postoperative complications observed in patients undergoing surgery for cystic hygroma (n=33)

Complications	n (%)
Injury to the marginal mandibular branch of the facial nerve	3 (9.09%)
Injury to the internal jugular vein	1 (3.03%)
Transient paralysis of the entire facial nerve (House-Brackmann grade IV)	1 (3.03%)
Neuropraxia of the spinal accessory nerve	1 (3.03%)
Wound dehiscence in the infra-auricular region requiring secondary suturing	2 (6.06%)

Recurrence of cystic hygroma was observed in four (12.12%) patients, whereas 28 (84.85%) patients did not show any recurrence. Among the recurrent cases, three patients had cavernous lymphangiomas located in the submandibular region, and one case had a macrocystic lesion. Management of recurrence involved re-excision in two cases and sclerotherapy in the remaining two cases. None of our patients presented with respiratory distress though CT scans in a few patients showed significant compromise of the airway in the pharynx due to lymphangioma pushing the pharyngeal walls.

## Discussion

Cystic hygroma or congenital lymphatic malformation results from the sequestration of lymphatic tissue during embryogenesis. It most commonly presents in the cervical region, as observed in our study where all 33 patients presented had cervical involvement. This finding is consistent with various existing literature. A study by Oulghoul et al. noted the cervical region as the predominant site of involvement, frequently extending into adjacent structures like the parotid and submaxillary areas, which pose a surgical challenge due to the proximity of critical structures such as the facial nerve and carotid bifurcation [11]. Similarly, Burezq et al. reported cervical involvement in 13 out of 14 cases, with some extending into the mediastinum, causing airway compression and carotid involvement [12].

In our study, it was observed that 22 (66.7%) were pediatric patients, with the majority aged between eight months and 13 years, consistent with the general observation that cystic hygromas typically present in infancy or early childhood. Interestingly, in our study, eight (24.2%) patients, though having a lesion since childhood, presented after the age of 13 years. This delayed presentation may reflect the limited health awareness and poor healthcare access in rural populations.

Our study had a higher incidence of 21 (63.6%) male patients contrasted by Oulghoul et al. who reported a female predominance with a sex ratio of 1.28:1, indicating potential regional variability [11]. Similarly, Zainine et al. documented a nearly equal male-to-female ratio (1.08), with 60% of cases presenting before the age of 10 and a peak frequency before two years of age [13].

Cystic hygromas are known to grow along tissue planes and can attain massive dimensions. Regarding lesion extent, 19 (57.8%) of our patients had extensive parapharyngeal involvement with further spread to the parotid in seven (21.21%) patients and submandibular in five (15.15%) patients and posterior triangle involvement in six (18.18%) patients. Extension below the clavicle was noted in two (6.06%) patients. Our observed parotid involvement is higher than the 9-28% range reported by Boardman et al., possibly due to larger lesion sizes or delayed presentation [14]. This higher prevalence aligns with findings from Oulghoul et al., who emphasized the complex surgical anatomy and difficult dissection while saving the nerve, with a higher chance of recurrence [11].

According to the de Serres staging, nine (27.27%) patients in our series had stage II lesions (suprahyoid involvement), and 24 (72.7%) patients had stage III involvement (suprahyoid and infrahyoid). This suggests that a significant proportion of patients presented with more extensive disease, posing increased challenges during surgical excision. In contrast, the study done by Hassanein documented that 32.5% of lesions were stage II and 30% were stage III [15]. Similarly, in a systematic review by Adams et al., they reported a frequency of 23.7% in stage II and 20.3% in stage III of the surgically managed patients [16].

Till the early 1980s, surgery was the only modality of treatment for large cystic hygromas with high complication rates [17,18]. Sclerotherapy was proposed as a treatment in early 1964 by Watson and McCarthy. Various sclerosing agents like ethanol, quinine, doxycycline, bleomycin, and OK-432 have been used in various institutions. OK-432 is considered best for sclerotherapy due to its lower morbidity and lower risk of damage to adjacent structures. This requires repeated sittings and was found to be unsuccessful in large lesions [19-23].

In our study, all 33 (100%) patients underwent surgery, and sclerotherapy was not used as the primary modality of treatment, as all our patients had large cystic hygromas; hence, the failure of sclerotherapy will make surgery more difficult due to repeated inflammation and fibrosis. Also, the patients from this socioeconomic background often show poor compliance, and the failure of sclerotherapy can cause fear and lead to discontinuation of treatment. In addition, a better sclerosing agent like OK-432 was not available in this region.

Complete excision was achieved in all the patients. Despite the complexity associated with large lesions and their proximity to vital neurovascular structures, our complication rate was low, and none were life-threatening. According to a study done by Adams et al., they noted a cranial nerve injury rate of 10.2% in surgical cases and an overall complication rate of 34.7% in the surgical group [16]. Charabi et al. reported an even higher complication burden, with 44% of patients experiencing functional impairments and 36% expressing cosmetic dissatisfaction [24]. Okazaki et al. documented lymphorrhea (27%), nerve palsy (8%), infections (6%), and even airway obstruction post-surgery [25].

In our series, the common complications observed were transient marginal mandibular nerve paralysis in three (9.09%) patients, followed by complete facial nerve palsy (House-Brackmann grade IV) in one (3.03%) patient, spinal accessory nerve neuropraxia in one (3.03%) patient, internal jugular vein injury in one (3.03%) patient, and wound dehiscence in the infra-auricular region requiring secondary suturing in two (6.06%) patients. The patient who had paralysis of the entire facial nerve recovered completely in six months. She had cystic hygroma involving the parotid region and hence required extensive dissection of the nerve during surgery, which could have caused neuropraxia. The three (9.09%) patients who had transient paralysis of the marginal mandibular nerve recovered completely within four months. These findings were consistent with a study by Zainine et al., who reported five facial nerve injuries (three marginal mandibular and two transient peripheral facial palsies) that resolved completely [13]. Hassanein documented transient facial nerve paresis in six patients (14%) and reported a total complication rate of 25.5% [15].

On histopathological examination, cavernous lesions were found in 19 (57.5%) patients, and macrocystic lesions were found in 14 (42.4%) patients. This contrasts with the findings of Oulghoul et al., who reported a predominance of macrocystic lesions (11 macrocystic vs. two cavernous), and Zainine et al., who also observed a macrocystic predominance [11,13]. Hassanein similarly found 70% macrocystic cases, while microcystic and mixed types were 16% and 14%, respectively [15].

All our patients had a minimum follow-up of three years. Recurrence occurred in four (12.12%) patients. Two (6.06%) patients had extensive cervical cystic hygroma extending to the parotid region and developed recurrence in the pre-auricular region after 18 months of surgery, for which re-excision was done. Two (6.06%) patients had recurrence in the floor of the mouth three years after surgery, and they opted for sclerotherapy using tetracycline. Similarly, a study by Oulghoul et al. documented one recurrence nine months postoperatively in the left jugal region, requiring reoperation [11]. Zainine et al. also reported a recurrence following the incomplete excision of a lesion extending into the floor of the mouth [13]. Charabi et al. reported the highest recurrence rates, up to 50%, and documented that suprahyoid involvement was significantly associated with residual or recurrent disease [24].

These comparative results emphasize that while surgical excision offers definitive treatment, especially in extensive lesions, it is associated with a notable risk of complications, particularly nerve injuries. However, with meticulous technique and appropriate preoperative planning, these risks can be minimized. In resource-limited settings, where access to effective sclerosing agents and consistent follow-up are inadequate, surgery remains a practical and effective first-line approach. Long-term follow-up is essential to detect and manage recurrence early, especially in cavernous and deeply located lesions.

Limitations

It is a retrospective analysis based on medical records, which may be prone to documentation bias. Being a single-institution study, the findings may not be generalizable to a larger population. The sample size was relatively small, limiting the statistical power of the analysis. Additionally, a comparative evaluation with sclerotherapy could not be performed due to poor treatment compliance among patients and the non-availability of advanced sclerosing agents like OK-432 in this region. These factors may affect the overall interpretation and applicability of the results in broader clinical settings.

## Conclusions

Cystic hygromas are lymphatic malformations encountered in the head and neck region. Due to its substantially large size, surgical excision was the mainstay of treatment for these malformations but is challenging due to its proximity to important neurovascular structures in the neck. However, meticulous discussion can minimize complications. Sclerotherapy is effective in treating these malformations but may require repeated sittings and cannot be done in large lesions. If cystic hygroma is completely excised, recurrences are uncommon.
